# Relationship between Admission Temperature and Risk of Cerebral Palsy in Infants Admitted to Special Care Unit in a Low Resource Setting: A Retrospective Single-Center Study

**DOI:** 10.3390/children9030352

**Published:** 2022-03-03

**Authors:** Chiara Guadagno, Francesco Cavallin, Luca Brasili, Donald Micah Maziku, Dionis Erasto Leluko, Gaetano Azzimonti, Giovanni Putoto, Andrea Pietravalle, Daniele Trevisanuto

**Affiliations:** 1 Doctors with Africa CUAMM, Dar es Salaam 23447, Tanzania; chiara100@hotmail.it (C.G.); l.brasili@cuamm.org (L.B.); g.azzimonti@cuamm.org (G.A.); 2 Independent Statistician, 36020 Solagna, Italy; cescocava@libero.it; 3 St. John of the Cross, Tosamaganga Council Designated Hospital, Tosamaganga 51201, Tanzania; dmaziku@yahoo.com (D.M.M.); dionisleluko97@gmail.com (D.E.L.); 4 Doctors with Africa CUAMM, 35121 Padua, Italy; g.putoto@cuamm.org; 5 Department of Woman’s and Child’s Health, University of Padua, 35128 Padua, Italy; daniele.trevisanuto@unipd.it

**Keywords:** birth, temperature, hypothermia, cerebral palsy, low-resource setting, neonate, neurological outcome

## Abstract

Background: Deviations from normothermia affect early mortality and morbidity, but the impact on neurodevelopment of the survivors is unclear. We aimed to investigate the relationship between neonatal temperature at admission and the risk of cerebral palsy (CP) at one month of age in a low-resource setting. Methods: This retrospective study included all inborn neonates admitted to the Special Care Unit of Tosamaganga Hospital (Tanzania) between 1 January 2019 and 31 December 2020. The neurological examination at one month of age was performed using the Hammersmith method. The relationship between the admission temperature and the risk of CP was investigated using logistic regression models, with temperature modeled as the non-linear term. Results: High/moderate risk of CP was found in 40/119 (33.6%) of the neonates at one month of age. A non-linear relationship between the admission temperature and moderate/high risk of CP at one month of age was found. The lowest probability of moderate/high risk of CP was estimated at admission temperatures of between 35 and 36 °C, with increasing probability when departing from such temperatures. Conclusions: In a low-resource setting, we found a U-shaped relationship between the admission temperature and the risk of CP at one month of life. Expanding the analysis of the follow-up data to 12–24 months of age would be desirable in order to confirm and strengthen such findings.

## 1. Introduction

About 2.4 million neonates still die every year globally, with a greater risk in their first day of life [1]. As neonatal temperature early after birth seems to play a crucial role, the World Health Organization (WHO) guidelines focused on thermal control since 1993, in order to support health care providers in thermal management and prevention of thermal loss [2]. Deviations from normothermia have been demonstrated to be important contributors to neonatal morbidity and mortality in both high- and low-resource settings [3,4]. Thermal control at birth is a critical challenge in low-resource settings, where proper thermal care is often inadequate and thermal stability is underestimated in management protocols [5,6,7,8]. Despite WHO recommendations, deviations from normothermia remain a public health issue in low-resource settings, where it is often under-recognized, under-documented, and poorly managed [8,9]. Beyond early mortality and morbidity, it is reasonable to suspect that deviations from normothermia may also impact the neurodevelopment of survivors [10]. Cerebral palsy (CP) is the most frequent physical disability in children, with decreasing prevalence in high-income countries and uncertain rates in low/middle-income countries [11]. Although the diagnosis of CP usually occurs at 12–24 months of age, it can be made before six months of age [11]. This is desirable given the therapeutic opportunities during the period of greatest brain development and neuroplasticity, but effective implementation is hampered by inadequate diagnostic equipment in low-income countries. In such settings, alternative diagnostic tools can provide affordable and reliable assessment of neurological damage [11]. This study aimed to investigate the relationship between neonatal temperature at admission and risk of CP at one month of age in a low-resource setting. Furthermore, the relationship between the admission temperature and mortality was also investigated in order to strengthen the considerations of the findings.

## 2. Materials and Methods

### 2.1. Study Design and Setting

This is a retrospective study on the relationship between neonatal temperature at admission and risk of CP at 1 month of age. The study was performed at the Special Care Unit (SCU) of Tosamaganga Hospital, which is a district designated hospital situated in the district of Iringa, Tanzania. This is a referral facility for a geographical area that covers about 260,000 people for major obstetric emergencies and has also a SCU offering basic intensive care, such as intravenous therapies, phototherapy, and oxygen supplementation without non-invasive respiratory support and mechanical ventilation. About 3000 deliveries and 500 admissions to the SCU occur every year at Tosamaganga Hospital. Moreover, since January 2019, a neonatal follow-up clinic has been available to check clinic well-being, growth, and neurological development for all babies discharged from the SCU during their first year of life.

### 2.2. Patients

All neonates admitted to the SCU of Tosamaganga Hospital between 1 January 2019 and 31 December 2020 were evaluated for inclusion in this study. Exclusion criteria were (i) outborn neonates, (ii) being admitted to the SCU after the first day of life, and (iii) missing data about body temperature at admission.

### 2.3. Data Collection

Due to the retrospective design, relevant information was collected in an anonymized dataset from hospital charts. When a baby was admitted to the SCU, the attending nurse measured the axillary temperature with a digital thermometer (C202; Terumo, Tokyo, Japan), and replicated the measurement if the temperature was <35 °C or >39 °C. According to WHO indications, temperatures in the 36.5–37.5°C range were classified as normothermia, >37.5 °C as hyperthermia, 36–36.4 °C as mild hypothermia, and <36 °C as severe/moderate hypothermia [12]. Admission diagnosis was made according to clinical assessment due to limited availability of laboratory and instrumental exams. Birth asphyxia was defined as 5-min Apgar Score of < 7. Respiratory distress was diagnosed in presence of signs of increased work of breathing (measured by the Silverman Anderson Score) and/or hypoxemia with need for oxygen supplementation. Early- or late-onset sepsis was diagnosed in presence of clinical signs (i.e., fever, hypotonia, irritability) within or after the first 7 days of life. Hypoglycemia was defined as blood glucose of < 2.6 mmol/L at any time. Jaundice was diagnosed according to the Kramer’s rule [13]. Weight loss was defined as body weight loss of >10%. Skin infection included abscess and omphalitis.

### 2.4. Neurological Examination

The neonatal follow-up program at Tosamaganga Hospital is scheduled at one, three, six, nine, and twelve months of corrected age for preterm infants and chronologic age for term infants. In this study, we chose to focus on the follow-up at 1 month of age, due to the high rate of lost to follow-up at later ages. As in many low-resource settings, the SCU lacked expensive diagnostic equipment (such as magnetic resonance imaging) and personnel trained in neurological examination. Hence, we identified affordable and reliable tools for neurological assessment, which were not time consuming or requiring specialized doctors. The neurological examination was performed using the standardized criteria provided by the Hammersmith method. We applied the Hammersmith Neonatal Neurological Examination (HNNE) by Spittle et al. [14], who used optimality scores between 10th–90th centile to define low risk, scores between 5th–10th, or 90th–95th, to define medium risk, and scores below 5th, or above 95th, to define high risk. Posters with images of neurological items by age were created and made available in the room where the examinations took place. The neurological examination was performed by healthcare providers with 6 month of on-the-job training (CG and LB) and each infant was classified at low risk, medium risk, or high risk of CP, as reported in Table 1 [14].

### 2.5. Statistical Analysis

Continuous data were summarized as median and interquartile range (IQR), and categorical data as number and percentage. The relationships between admission temperature and (i) mortality and (ii) risk of CP were investigated with logistic regression models where temperature was modeled with first-, second-, and third-order polynomials, and with restricted cubic splines. Furthermore, multivariable logistic regressions were applied to assess the effect of admission temperature on mortality (adjusting for birth weight, Apgar score at 5 min, major malformations, and meconium-stained amniotic fluid) and risk of CP (adjusting for birth weight and Apgar score at 5 min). The adjusting variables were chosen among the clinically relevant variables and according to the number of neonates with the event, while gestational age could not be included because it was largely missing. All tests were two-sided and a *p*-value less than 0.05 was considered statistically significant. Statistical analysis was performed using R 4.1 (R Foundation for Statistical Computing, Vienna, Austria) [15].

## 3. Results

**Figure 1:**
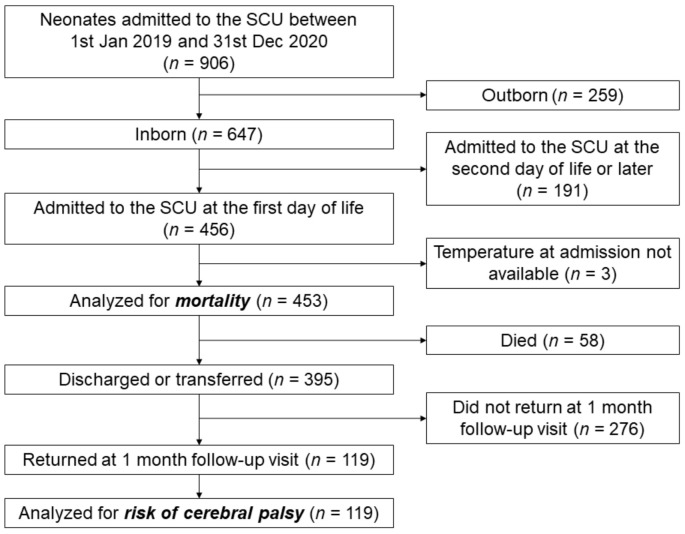
Flowchart of patient selection for analysis of mortality and risk of cerebral palsy among inborn neonates who were admitted on their day of birth to the Special Care Unit of Tosamaganga Hospital between 1 January 2019 and 31 December 2020.

A total of 906 newborns were admitted to the SCU of Tosamaganga Hospital between 1 January 2019 and 31 December 2020. Of them, 450 were excluded from the analysis because they were outborn (*n* = 259) or were admitted after their day of birth (*n* = 191). A further three infants were excluded due to missing temperature data at admission (Figure 1). The remaining 453 newborns were included in this analysis and their characteristics are reported in Table 2. Information on the gestational age was largely missing (420/459, 91.5%). The median temperature at admission was 35.4 °C (IQR 34.7–36.1 °C; min 30.6 °C, max 37.5 °C). Severe/moderate hypothermia was reported in 316 of the neonates (69.7%), mild hypothermia in 66 (14.6%), normal temperature in 71 (15.7%), and no infants with a temperature higher than 37.5 °C were observed. The treatments included antibiotics (301 neonates, 66.4%), anticonvulsant (48 neonates, 10.6%), and aminophylline/caffeine (78 neonates, 17.2%). Oxygen therapy was administered to 399 neonates (88.1%) for a median of two days (IQR 1–5). IV fluids were administered to 323 neonates (71.3%) for a median of five days (IQR 3–8). Phototherapy was offered to 67 neonates (14.8%).

**Figure 2:**
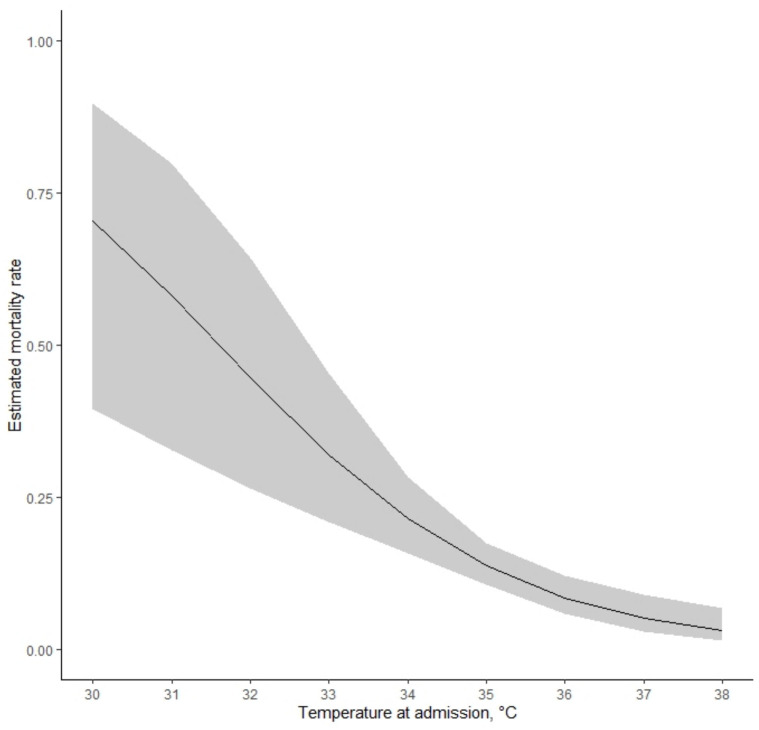
Estimated mortality rate according to neonatal temperature at admission as modeled with first-order polynomial. Shaded areas represent bootstrap 95% confidence intervals.

After a median length of stay of six days (IQR 4–11), 58 neonates died (12.8%) while 390 were discharged (86.1%) and five were transferred to other health facilities (1.1%). The analysis suggested modelling the relationship between mortality and admission temperature with first-order polynomial (*p* < 0.0001), showing the highest mortality risk for decreasing temperature (Figure 2). At the multivariable analysis, temperature at admission was not an independent predictor of mortality (*p* = 0.46), adjusting for birth weight (*p* < 0.0001), Apgar score at 5 min (*p* = 0.0008), major malformations (*p* < 0.0001), and meconium-stained amniotic fluid (*p* = 0.56).

**Figure 3:**
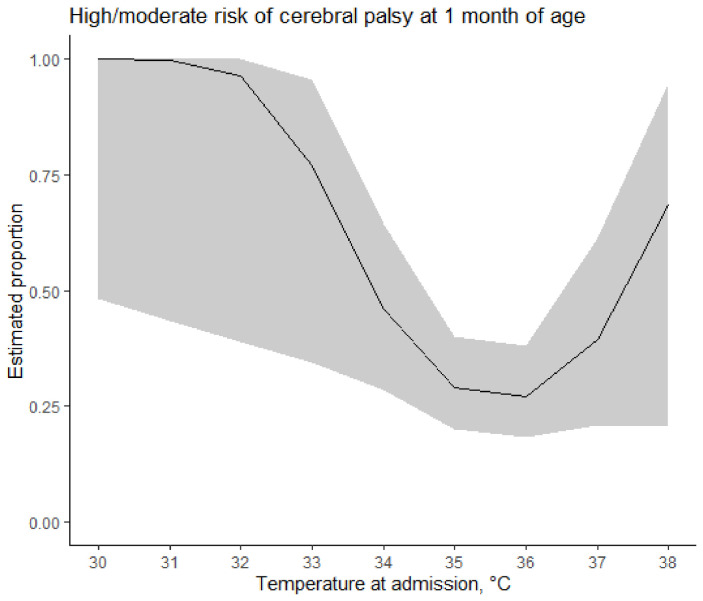
Estimated proportion of high/moderate risk of cerebral palsy at 1 month of age. Shaded areas represent bootstrap 95% confidence intervals.

Overall, 119 out of 395 neonates (30.1%) returned at the follow-up visit at one month of age. The neurodevelopmental assessment suggested low risk of CP in 79 neonates (66.4%), moderate risk in 31 (26.0%), and high risk in nine (7.6%). The analysis suggested modelling the relationship between high/moderate risk of CP at one month of age and the admission temperature with second-order polynomial (*p* = 0.04), showing the lowest probability between 35–36 °C and increasing probability when departing from the 35–36 °C range (Figure 3). At the multivariable analysis, the temperature at admission was still associated with risk of CP (*p* = 0.04), adjusting for birth weight (*p* = 0.002) and Apgar score at 5 min (*p* = 0.18).

## 4. Discussion

Our findings showed a small proportion of neonatal normothermia in a low-resource setting and a non-linear relationship between the admission temperature and the risk of CP at one month of life. To our knowledge, this is the first study to explore the variation in the risk of CP across a wide range of admission temperatures and to suggest a non-linear relationship between the admission temperature and the risk of CP in a low-resource setting. Our results extend previous reports on a U-shaped relationship between admission temperature and neonatal morbidity and mortality in high- and low-resource settings [3,4], suggesting a U-shaped relationship between the admission temperature and the risk of CP in survivors at one month of life. The study also has some limitations that should be considered by the reader. First, the retrospective design does not allow us to draw any causal relationships. Second, the compliance to follow-up visits was partial, hence limiting the representativeness of the findings for the original sample of the discharged neonates. Third, the short duration of the follow-up assessment only allows speculations about the clinical confirmation of the long-term neurological status. Finally, we applied the HNNE by Spittle et al. [14], hence we estimated the category of gestational age (moderate preterm infants, late preterm infants, term infants) by using the relationship between 50^th^ percentile of birthweight and gestational age. Literature offers heterogeneous information about neonatal temperature at admission and neurological outcome. Previous studies mainly focused on hypothermia and intraventricular hemorrhage (IVH)—as high-grade IVH is an important cause of severe cognitive and motor neurologic impairment—in very preterm infants, and reported conflicting results. Laptook et al. [16], Wilson et al. [17], Chang et al. [18], and Ng’eny et al. [19] did not find any significant associations between admission temperature and severe IVH in very preterm infants in the United States, Europe, Taiwan, and South Africa. Of note, Chang et al. did not find any associations also with two-year neurodevelopmental impairment [18]. On the other hand, Miller et al. [20] and Yu et al. [21] reported an increased risk of IVH in hypothermic very preterm infants in the United States and China. In addition, Lyu et al. [3] showed a U-shaped relationship between admission temperature and severe neurological damage (IVH or periventricular leukomalacia) in very preterm infants in Canada. Our data suggest a similar U-shaped relationship between the admission temperature and moderate/high risk of CP during early follow-up. The lowest probability of moderate/high risk of CP was estimated at the admission temperature of between 35–36 °C, with increasing probability when departing from such temperature. However, the reader should be aware that lower admission temperature was associated with higher in-hospital mortality risk, but the analysis of risk of CP could be performed in the subgroup of survivors who returned at the one-month follow-up visit, thus preventing us from drawing a combined consideration. Overall, our results convey a similar message about such U-shaped relationship, although there are some differences in terms of the timing of the assessment (one month vs. before discharge), outcome measure (CP vs. IVH), population (term + preterm vs. very preterm infants), temperature range (30.6–37.5 °C vs. 32–41 °C), and study setting (low- vs. high-resource). We believe that such aspects may explain the slight difference in the estimated temperature range with the lowest risk of adverse neurological outcome (35–36 °C vs. 36.8 °C). In sub-Saharan Africa, the incidence of postnatal hypothermia is very high, with figures ranging from 32 to 85% [5]. In our study, we found a large proportion (84.3%) of neonates who were admitted with hypothermia, thus highlighting that the prevention of postnatal thermal loss is still an unsolved major challenge in these settings. In addition, the high proportion of birth asphyxia can be explained by the patient selection (inborn neonates who were admitted at the first day of life) and the referral role of the Tosamaganga Hospital. Of note, only one in three of the discharged neonates returned for the follow-up visits. In low-resource settings, this situation has been commonly reported in other pediatric situations, such as immunization or malnutrition [22,23]. Beyond limiting the representativeness of the findings about risk of CP, this information calls for actions to investigate the reasons for non-adherence to follow-up visits and to plan adequate interventions in order to overcome the barriers to adherence [24]. In addition, expanding the analysis of the follow-up data to 12–24 months of age would be desirable in order to strengthen the findings and confirm the U-shaped association between the admission temperature and the risk of CP.

## 5. Conclusions

In a low-resource setting, we found a U-shaped relationship between the admission temperature and the risk of CP at one month of life. Beyond the study limitations, these findings add a special alert about the possible association between the admission temperature and the risk of adverse neurological outcomes among newborns in low-resource settings. As disabilities have a large burden in low-resource settings, enhancing the efforts to prevent all of the identified associated conditions, such as deviations from normothermia, could be desirable.

## Figures and Tables

**Table 1:** Classification of risk of cerebral palsy according to the Hammersmith Neonatal Neurological Examination (HNNE).

Risk	HNNE
Low risk	27–33
Medium risk	25.4–26.9 or 33.1–33.5
High risk	<25.4 or >33.5

**Table 2:** Characteristics of inborn neonates who were admitted on their day of birth to the Special Care Unit of Tosamaganga Hospital between 1 January 2019 and 31 December 2020 and those who attended the 1-month follow-up.

	Admitted Neonates (*n* = 453)	Newborns at 1 Month Follow-Up (*n* = 119)
Males	237 (52.3)	65 (54.6)
Birth weight, grams ^a^	2750 (1940–3180)	2610 (1795–3055)
Birth weight:		
Normal birth weight (≥2500 g)	269 (59.4)	62 (52.1)
Low birth weight (1500–2499 g)	130 (28.7)	43 (36.1)
Very low birth weight (1000–1499 g)	42 (9.3)	14 (11.8)
Extremely low birth weight (<1000 g)	12 (2.6)	0 (0.0)
Temperature at admission, °C ^a^	35.4 (34.7–36.1)	35.4 (24.8–35.9)
Temperature at admission:		
36.5–37.5 °C	71 (15.7)	16 (13.5)
36–36.5 °C	66 (14.6)	13 (10.9)
<36 °C	316 (69.7)	90 (75.6)
HIV-positive mother	35/450 (7.8)	13/118 (11.0)
Maternal VDRL ^b^	7/438 (1.6)	1/117 (0.9)
Apgar score at 1 min ^a^	5 (3–7)	5 (2–8)
Apgar score at 5 min ^a^	7 (5–10)	8 (5–10)
PROM ^c^	86 (19.0)	22 (18.5)
Meconium-stained amniotic fluid	133 (29.4)	34 (28.6)
Maternal fever	12 (2.6)	2 (1.7)
Dexamethasone:		
None	414 (91.4)	99 (84.2)
Complete cycle (24 mg)	26 (5.7)	14 (11.8)
Incomplete cycle (12 mg)	13 (2.9)	6 (5.0)
Mode of delivery		
Spontaneous vaginal delivery	221 (48.8)	63 (52.9)
Assisted vaginal delivery	42 (9.3)	11 (9.3)
Caesarean section	190 (41.9)	45 (37.8)
Twin pregnancy	61/452 (13.5)	24 (20.2)
Birth asphyxia	171 (37.7)	49 (41.2)
Respiratory distress	308 (68.0)	83 (69.7)
Early-onset sepsis	22 (4.9)	6 (5.0)
Late-onset sepsis	18 (4.0)	5 (4.2)
Hypoglycemia	20/428 (4.7)	7/114 (6.1)
Jaundice	85 (18.8)	29 (24.4)
Weight loss	10 (2.2)	2 (1.7)
Skin infection	14 (3.1)	8 (6.7)
Major malformations or chromosomopathies ^d^	19 (4.2)	2 (1.7)

Data were summarized as *n* (%) or ^a^ median (IQR). Definitions are provided in Methods section. HIV: human immunodeficiency virus. PROM: prolonged rupture of membranes. VDRL: venereal disease research laboratory test. ^b^ VDRL was treated in 5/7 mothers. ^c^ PROM prophylaxis in 18/88 mothers. ^d^ Major malformations included cardiac heart disease (*n* = 9), club feet (*n* = 3), conjoined sibling (*n* = 2), cranial malformation (*n* = 1), Down syndrome (*n* = 1), myelomeningocele and encephalocele (*n* = 1), imperforate anus (*n* = 1), arthrogryposis (*n* = 1) and hypospadias (*n* = 1).

## Data Availability

The datasets used and/or analyzed during the current study are available from the corresponding author on reasonable request.
